# Retraction of: Transcription factor Liv4 is required for growth and pathogenesis of *Cryptococcus neoformans*

**DOI:** 10.1093/femsyr/foad042

**Published:** 2023-10-18

This is a retraction of Jiu Yi, Junjun Sang, Jingyu Zhao, Lei Gao, Yali Yang, Lei Yan, Chao Zhang, Weihua Pan, Guizhen Wang, Wanqing Liao, Transcription factor Liv4 is required for growth and pathogenesis of *Cryptococcus neoformans, FEMS Yeast Research*, Volume 20, Issue 3, 11 May 2020, https://doi.org/10.1093/femsyr/foaa015.

The last-named author contacted the journal editor in May 2023, after being alerted to concerns with duplicated images in Figure 2, which had been posted on PubPeer in November 2021 (https://pubpeer.com/publications/4D828B96E005A6D0D0A2A74312D5B4). That individual asserted they had not been aware of the article, noted inconsistencies with the data presented, did not endorse the research results reported, and stated they had consulted the other named authors, who collectively agreed the article should be retracted. The journal subsequently attempted to contact the other named authors directly, but none of them were responsive. The journal is ultimately retracting this article, because the editor no longer believes the data presented in Figure 2 provides confidence in the construction of the *liv4*Δ mutant, which anchors the study.

